# Surgical Treatment of Primary Localized Cutaneous Nodular Amyloidosis of the Nose: Resection and Reconstruction With a Nasolabial Flap

**DOI:** 10.7759/cureus.99878

**Published:** 2025-12-22

**Authors:** Daisuke Hirao, Tomoki Himejima, Michika Fukui, Masakatsu Hihara, Natsuko Kakudo

**Affiliations:** 1 Department of Plastic and Reconstructive Surgery, Kansai Medical University, Hirakata, JPN; 2 Department of Plastic and Reconstructive Surgery, Yao Municipal Hospital, Yao, JPN

**Keywords:** amyloidosis, cutaneous, nasolabial flap, nose, plcna

## Abstract

Primary localized cutaneous nodular amyloidosis (PLCNA) is a rare form of amyloidosis limited to the skin. A 39-year-old woman had noticed a tumor on her right nasal ala for five years. She presented it to our hospital after developing a similar lesion on her right cheek. Histopathological examination following surgical excision revealed AL amyloidosis (κ-light chain type), ruling out systemic amyloidosis and confirming the diagnosis of PLCNA. The nasal lesion was excised and reconstructed with a nasolabial flap, resulting in excellent aesthetic and functional outcomes. No recurrence has been observed during a 21-month follow-up. PLCNA in young adults is rare, and long-term follow-up is crucial due to the potential for systemic progression. This case highlights the importance of accurate diagnosis and the benefits of local flap reconstruction in cosmetically sensitive areas.

## Introduction

Amyloidosis refers to a group of disorders characterized by the abnormal deposition of proteins that affects tissue architecture and function [1]. It is broadly classified as systemic or localized, depending on the distribution of these deposits.

When deposition is confined to the skin, the condition is termed primary localized cutaneous amyloidosis. It is defined by an absence of internal organ involvement and is traditionally divided into three clinicopathologic subtypes: macular, lichenoid, and nodular [2]. Macular and lichenoid types are relatively common and typically present as pruritic pigmented patches or hyperkeratotic papules. In contrast, primary localized cutaneous nodular amyloidosis (PLCNA) is considered the rarest and most unusual variant. Histologically, it is characterized by dermal and subcutaneous deposits of amyloid derived from immunoglobulin light chains, which are often associated with plasma cell infiltration [3,4].

Herein, we present a rare case of PLCNA localized to the nasal and cheek regions. The lesion was successfully excised, and the area was reconstructed with a nasolabial flap, providing satisfactory cosmetic and functional outcomes.

## Case presentation

A 39-year-old woman first noticed a skin lesion on the right nasal ala five years earlier. She underwent cryotherapy at a dermatology clinic, but there was no improvement. A skin biopsy revealed amyloid deposition, resulting in a diagnosis of amyloidosis. Laser therapy was attempted, but it was unsuccessful, and the nasal lesion gradually enlarged. Subsequently, a similar lesion developed on the right cheek. The patient then presented to our department with tumors on the right nasal ala and cheek. Her past medical and family histories were unremarkable.

**Figure 1:**
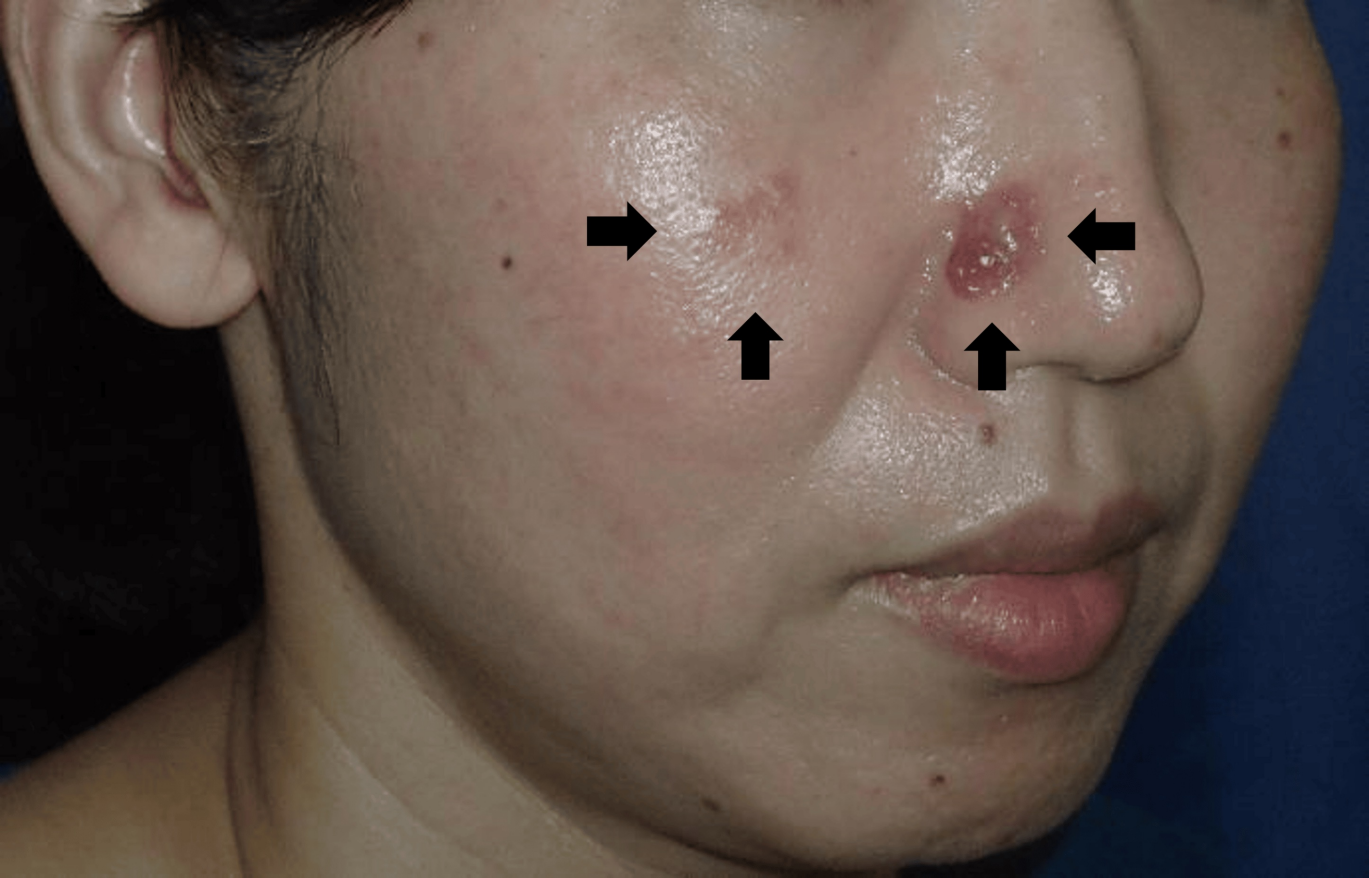
Clinical features of the patient. A 17 × 14 mm reddish nodule on the right nasal ala and a 14 × 12 mm reddish papule on the right cheek.

The lesion on the right nasal ala measured 17 × 14 mm and appeared as a reddish nodular tumor. The lesion on the right cheek measured 14 × 12 mm and presented as a pale red papule (Figure 1). Both lesions were excised under local anesthesia with a 1-mm peripheral margin. The deep margin was set at the level of the muscle for the nasal ala lesion and at the level of the subcutaneous fat for the cheek lesion.

**Figure 2:**
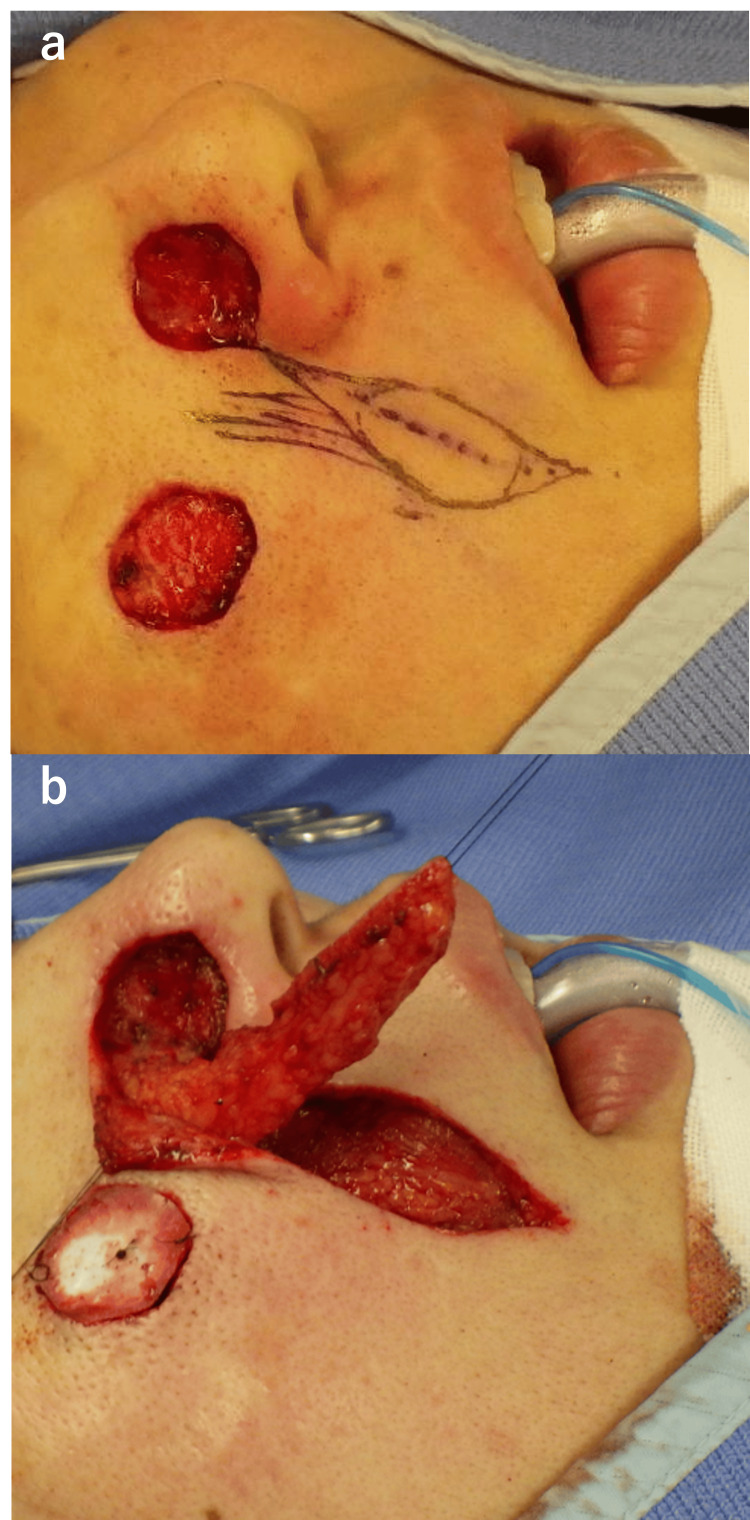
Reconstruction of the nasal ala. (a) Design of the nasolabial flap. (b) Elevation of the nasolabial flap.

Additional resection was performed on both lesions. The horizontal margin was extended by 3 mm for further excision. For the nasal ala tumor, resection was performed above the perichondrium. The nasal ala defect was reconstructed with a nasolabial fold flap, and the cheek lesion was covered with artificial dermis (Figures 2a, 2b).

**Figure 3:**
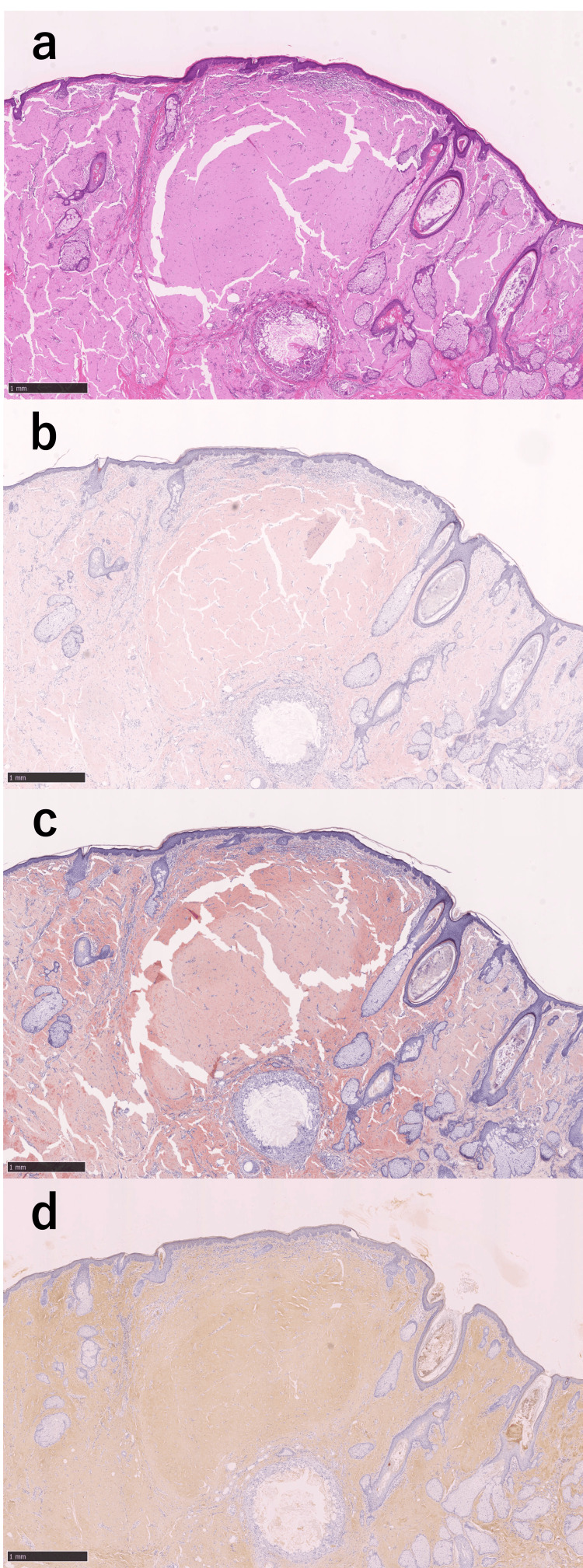
Histopathological features of amyloidosis (bar: 1 mm for all). (a) Amorphous eosinophilic deposits were observed in the superficial to deep dermis (hematoxylin and eosin). (b) Amyloid fibrils stained light red with Congo red. (c) Amyloid fibrils stained orange-red with direct fast scarlet. (d) Immunohistochemical staining demonstrated positivity only for the anti-kappa light chain antibody.

A histopathological examination using hematoxylin and eosin stains revealed amorphous eosinophilic deposits extending from the superficial to the deep dermis of the nasal ala lesion. Congo red staining revealed pale red amyloid fibrils, while direct fast scarlet staining highlighted orange-red deposits. Immunohistochemical staining revealed positivity only for the anti-kappa light chain antibody within the lesion (Figures 3a-3d).

The nasal ala tumor had positive horizontal and deep margins, while the cheek lesion had positive horizontal margins only. To further examine the possibility of systemic amyloidosis, we consulted with hematologists and rheumatologists. We performed comprehensive evaluations, including a complete blood count, metabolic panel, electrocardiography, serum M protein test, urinary Bence-Jones protein test, and anti-SSA/SSB antibody test (for diagnosing Sjögren’s syndrome). All results were within normal limits. Based on the test results and lesion characteristics, a diagnosis of PLCNA was made.

**Figure 4:**
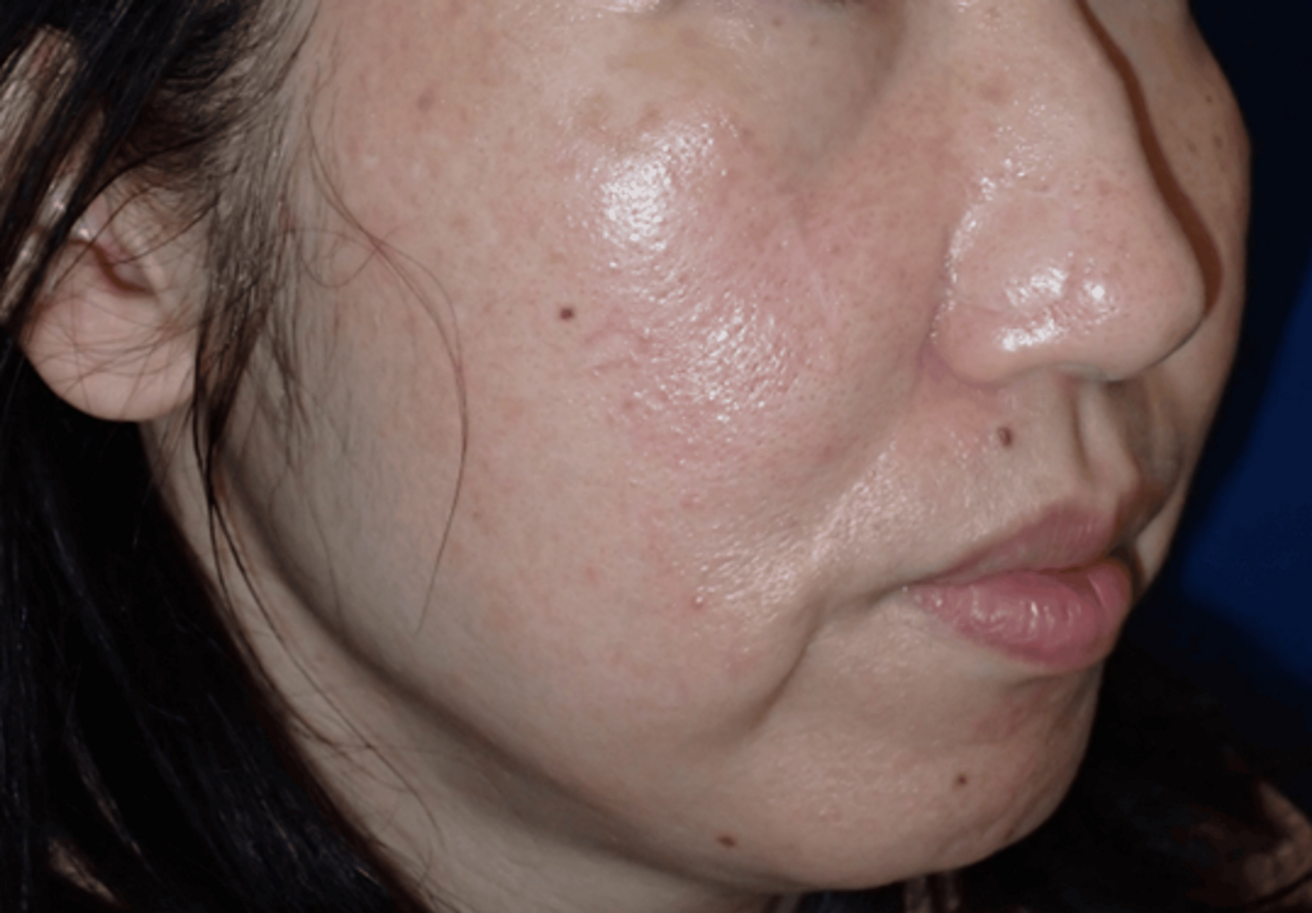
Postoperative view. No recurrence has been observed 21 months after nasolabial flap reconstruction.

At 21 months postoperatively, no recurrence of the tumor has been observed, and the patient remains satisfied with the aesthetic outcome (Figure 4). Annual follow-up was performed to detect local recurrence or the development of lesions at other sites, and systemic involvement was monitored in collaboration with the departments of hematology and rheumatology.

## Discussion

PLCNA is the rarest subtype of primary localized cutaneous amyloidosis, accounting for approximately 1.5% of all cases [5]. Nodular amyloidosis was first described by Gottron [6]. Clinically, it manifests as solitary or multiple yellow, brown, or red nodules, ranging from 0.5 to 7 cm in size, typically located on the extremities, trunk, neck, or acral regions [7]. PLCNA lesions may be solitary or multiple and most commonly occur in the acral region, head, neck, and extremities [3]. The mean age of onset for nodular primary localized cutaneous amyloidosis is 55 years (range, 33-86 years), with no significant differences in sex or race [4]. No definitive risk factors have been identified [8]. The mechanism underlying amyloid deposition is still unknown [8]. 

A histopathological examination is indispensable for a definitive diagnosis. In the nodular subtype, the deposited amyloid material is derived from immunoglobulin light chains (AL type), which are produced by dermal plasma cell infiltrates. This material can be identified using Congo red staining or immunohistochemistry for κ and λ light chains. In other forms of primary cutaneous amyloidosis, the amyloid fibrils originate from keratinocytes; therefore, differentiation from the nodular type is possible [4]. However, these features are virtually indistinguishable from those observed in systemic AL amyloidosis, highlighting the importance of differentiating between localized and systemic disease [2]. In this case, the patient was 39 years old, representing a relatively early onset. 

To diagnose PLCNA, it is crucial to rule out systemic amyloidosis because the histological features of PLCNA and the cutaneous manifestations of systemic AL amyloidosis are nearly identical. Recommended evaluations include a physical examination, complete blood count, metabolic panel, and assessment of serum M protein and urinary Bence-Jones protein to rule out an extraneous monoclonal plasma cell population. Electrocardiography may also be performed to rule out cardiac involvement. Negative results from these evaluations support a diagnosis of PLCNA. Furthermore, PLCNA has been associated with autoimmune diseases, including Sjögren’s syndrome, systemic lupus erythematosus, and systemic sclerosis, with Sjögren’s syndrome being most frequently described [4,9-11]. 

In this case, systemic disease was ruled out through consultations with hematologists and rheumatologists because physical examinations, blood tests, serum M protein, urinary Bence-Jones protein, anti-SS-A and anti-SS-B antibodies, and electrocardiography were all normal, resulting in the diagnosis of PLCNA. Notably, cases have been reported in which PLCNA progressed to systemic amyloidosis up to 23 years after the initial diagnosis, with reported progression rates ranging from 7% to 50% [12]. Recurrence is also not uncommon, even after complete excision [4]. Therefore, long-term follow-up is essential.

Due to its rarity, there is no standardized treatment for PLCNA [2]. Reported treatment modalities include surgical excision, CO₂ laser therapy, cryotherapy, and pharmacological approaches [3]. One report suggests that surgical excision is the most effective of these treatments [13]. In this case, the initial excision was performed with a 1-mm margin, as PLCNA is considered a benign tumor. Histopathological examination revealed positive margins; therefore, additional excision was undertaken to reduce the risk of local recurrence. Because PLCNA is benign, an optimal surgical margin has not been clearly established. While complete excision is important to prevent local recurrence, excessively wide margins may result in overtreatment. Treatment options for managing facial skin defects include primary closure, local flaps, skin grafting, and secondary healing with ointment therapy. Although skin grafting and secondary healing may be effective in certain situations, they often fail to provide satisfactory cosmetic outcomes due to differences in skin texture and color. In this case, the lesion was located on the nose, with only a small skin defect remaining after excision. From an aesthetic standpoint, reconstruction with a local flap was considered appropriate [14]. Local flaps use adjacent skin and often yield superior cosmetic outcomes. Among the various options for nasal reconstruction, we chose a nasolabial flap because it allows the donor site to align with natural skin folds. This technique produced favorable cosmetic results, with which the patient was highly satisfied. For small nasal alar defects, like the one in this case, the nasolabial flap represents an excellent reconstructive option.

## Conclusions

When diagnosing PLCNA in the nasal and cheek regions, it is crucial to rule out systemic involvement. Surgical excision is an effective treatment, and reconstruction with a local flap can result in favorable aesthetic and functional outcomes. However, long-term follow-up is necessary due to the risk of systemic progression.

## References

[1] Kendler M, Helbig D, Paasch U (2011). Nodular localized primary cutaneous amyloidosis and primary marginal zone B-cell lymphoma on the nose: treatment with microscopically controlled surgery. Int J Dermatol.

[2] Almukhlifi A, Ibrahim A, Hazazzi A, Almadani N (2025). Primary localized cutaneous amyloidosis presenting with tumor-like swelling in the lower extremity: a rare presentation. Cureus.

[3] Llamas-Molina JM, Velasco-Amador JP, De La Torre-Gomar FJ (2023). Localized cutaneous nodular amyloidosis in a patient with Sjögren’s syndrome. Int J Mol Sci.

[4] Kwon IJ, Yoo DS, Roh MR (2023). Primary localized cutaneous nodular amyloidosis on scalp successfully treated with excision. Ann Dermatol.

[5] Bellinato F, Rosina P, Sina S, Girolomoni G (2022). Primary nodular localized cutaneous amyloidosis of the scalp associated with systemic lupus erythematosus. Arch Rheumatol.

[6] Gottron VHA (1950). Amyloidosis cutis nodularis atrophicans diabetic. Dtsch Med Wochenschr.

[7] Dokic Y, Subrt P, Tschen J (2019). A rare presentation of nodular amyloidosis on the lower back. Cureus.

[8] Wu X, Zhao Z (2021). Primary localized cutaneous nodular amyloidosis presenting as lymphatic malformation: A case report. Open Life Sci.

[9] Llamas-Molina JM, Velasco-Amador JP, De la Torre-Gomar FJ (2023). Localized cutaneous nodular amyloidosis: a specific cutaneous manifestation of Sjögren’s syndrome. Int J Mol Sci.

[10] Yoneyama K, Tochigi N, Oikawa A (2005). Primary localized cutaneous nodular amyloidosis in a patient with Sjögren's syndrome: a review of the literature. J Dermatol.

[11] Summers EM, Kendrick CG (2008). Primary localized cutaneous nodular amyloidosis and CREST syndrome: a case report and review of the literature. Cutis.

[12] Woollons A, Black MM (2001). Nodular localized primary cutaneous amyloidosis: a long-term follow-up study. Br J Dermatol.

[13] Wang QX, Ye Q, Zhou KY (2025). Systematic review and meta-analysis of treatments and outcomes in primary localized cutaneous amyloidosis. Clin Exp Dermatol.

[14] Okazaki M, Hisatomi T, Sarukawa S (2006). Aesthetic upper lip reconstruction with vermilion submucosal-pedicle cross-lip flap. J Craniofac Surg.

